# Resistance of *Alternaria* spp. Causing Strawberry Black Spot to Boscalid in China

**DOI:** 10.3390/plants14131941

**Published:** 2025-06-24

**Authors:** Tao Li, Wenbin Yu, Ji Feng, Chengxin Mao, Hong Yu, Aichun Liu, Chuanqing Zhang

**Affiliations:** 1 Department of Plant Protection, College of Advanced Agricultural Sciences, Zhejiang A&F University, Hangzhou 311300, China; litaolitao1990@126.com (T.L.);; 2 Zhejiang Key Laboratory of Biology and Ecological Regulation of Crop Pathogens and Insects, Hangzhou 310013, China; 3 Research Institute for the Agriculture Science of Hangzhou, Hangzhou 310013, China

**Keywords:** strawberry black spot, boscalid, resistant, fitness cost, cross-resistance

## Abstract

Strawberry black spot, caused by *Alternaria* spp., is an emerging disease that threatens both leaves and fruits during strawberry growth and postharvest storage. This study investigated the boscalid sensitivity of 49 *Alternaria* isolates collected from symptomatic strawberry leaves. Boscalid has been widely used to control diseases in strawberry in China for several years. The EC_50_ values for the tested isolates ranged from 0.0884 to 266.3289 µg/mL, indicating that most isolates exhibited varying levels of resistance to boscalid based on resistance ratio values. A substitution of SDHC-H134R was detected from most high-resistance isolates. Fitness cost assessment revealed that highly resistant isolates had a reduced conidial germination rate; however, their mycelial growth and conidia production were increased. No significant virulence deficiency was observed, suggesting low fitness cost in resistant isolates. Furthermore, the highly resistant isolates exhibited positive cross-resistance to fluopyram and fluxapyroxad. Molecular docking analysis revealed that the SDHC-H134R mutation reduced the binding affinity between boscalid and mitochondrial complex II. These findings suggest that resistance management strategies, such as fungicide rotation or combinations of fungicides with different action modes, should be implemented to control strawberry diseases, minimizing the development of fungicide resistance and improving overall disease control efficacy.

## 1. Introduction

Strawberry (*Fragaria* × *ananassa*) is a globally important fruit crop, widely cultivated for its high market demand and nutritional value. It is favored by consumers due to its content of vitamin B, vitamin C, proteins and minerals [1]. In 2022, China became the biggest consumer and producer of strawberry in the world, with a total production of 398,200 tons and cultivation area of 147,500 hectares (http://zdscxx.moa.gov.cn:8080/nyb/pc/search.jsp, accessed on 15 September 2024). However, strawberry production is constrained by several factors during cultivation, transport and storage. These include mechanical damage, which reduces fruit quality and susceptibility to rot-causing fungi, which shortens shelf life [2,3]. Additionally, several pathogens can decrease strawberry yields by reducing active photosynthetic leaf area or causing wilt in strawberry plants during the growing season.

Throughout the growing period, various fungal species can infect strawberry and cause typical symptoms on the plants or fruits. Among these, *Fusarium oxysporum* and *Colletotrichum* species are the primary causal agent of seedling wilt, infecting the roots, petioles, or basal stems [4,5,6]; *Sphaerotheca aphanis* and *Botrytis cinerea* predominantly infect leaves and fruits, causing leaf blight and fruit rot, respectively [7,8]. Furthermore, *Alternaria* spp. can also reduce yield by causing black leaf spot, thereby reducing the active photosynthetic leaf area during the production period, or by causing fruit rot in the postharvest period, which reduces fruit quality [9,10,11]. Strawberry black spot, caused by *Alternaria alternata*, was first reported in Japan [12] and has since been found in Italy, Korea and Pakistan [13,14,15]. *Alternaria tenuissima* has also been reported in association with strawberry black spot [16]. In recent years, both *A. tenuissima* and *A. alternata* have been identified as causal agents of black leaf spot in strawberry, which is widespread in strawberry growing operations in Beijing and Dandong, China [9,11]. Although the damage caused by *Alternaria* spp. is generally not as severe as that caused by *B. cinerea*, *F. oxysporum* and *Colletotrichum* species, the control of strawberry black leaf spot warrants attention, particularly in areas where the disease has spread.

Chemical fungicides represent the primary method for controlling plant diseases in both field and greenhouse. Several studies have evaluated the efficacy of fungicides against *Alternaria* spp. on various hosts. For example, mefentrifluconazole has demonstrated excellent inhibition activity against *A. tenuissima* causing tomato early blight, and boscalid has shown high efficacy against *Alternaria* spp. causing brown spot of citrus and pear [17,18,19]. However, limited research has focused on chemical control options for the pathogens causing strawberry black spot. Difenoconazole, propiconazole and picoxystrobin have been investigated and shown to inhibit mycelial growth of *A. tenuissima* associated with strawberry black spot [20]. Furthermore, some biological agents, such as arbuscular mycorrhizal fungi and an isolate of *Meyerozyma guilliermondii* SQUCC-33Y, have been reported to suppress strawberry black spot caused by *A. alternata* [21,22]. Boscalid, a significant SDHI fungicide with a substantial market share, has been registered for several years in China for the management of strawberry disease gray mold, which is caused by *B. cinerea* (http://www.chinapesticide.org.cn/). Boscalid resistance in *B. cinerea* has been reported in several regions of China [23,24]. However, information regarding the sensitivity to boscalid of *Alternaria* spp., causing strawberry black spot, is limited.

Therefore, the objectives of this study were to (i) investigate the sensitivity of *Alternaria* spp., the causal pathogen of strawberry black spot, to boscalid; (ii) determine whether *Alternaria* spp. have developed resistance to boscalid, a fungicide commonly used in strawberry disease control; and (iii) investigate the resistance mechanism and fitness cost associated with resistant isolates. This research will provide valuable information and contribute to the development of improved management strategies for strawberry black spot.

## 2. Results

### 2.1. Sensitivity to Boscalid

**Figure 1:**
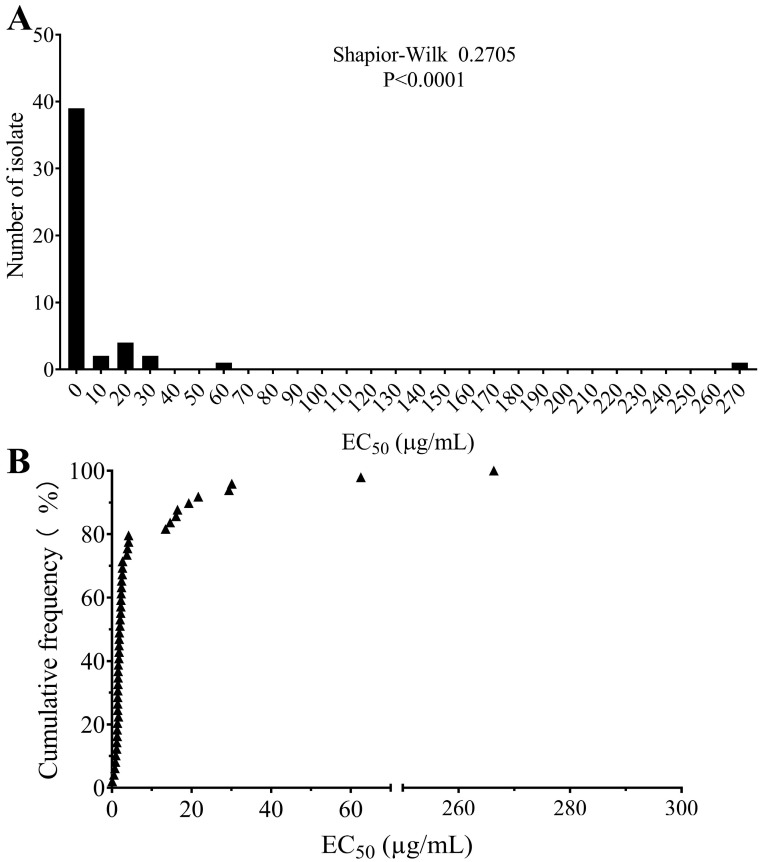
Distribution of EC_50_ (N = 49). (**A**) Frequency distribution of EC_50_ values. (**B**) Cumulative frequency distribution of EC_50_ values.

In the preliminary test, five randomly selected isolates exhibited varying degrees of sensitivity to boscalid (Figure S1) and the concentrations used in the sensitivity assays were adjusted accordingly. The EC_50_ values of all 49 isolates ranged from 0.0884 to 266.3289 µg/mL, with a mean of 11.5190 ± 5.5348 µg/mL. The distribution of EC_50_ values was non-unimodal (Shapiro–Wilk = 0.2175, *p* < 0.0001). A subset of isolates exhibited significantly reduced sensitivity to boscalid, with EC_50_ values exceeding 100 µg/mL (Figure 1). Resistance ratio value (RRV) was calculated based on the EC_50_ values and resistance levels were classified according to the criteria described by Liu et al. [25]. Based on these criteria, three isolates (6.12%) were classified as sensitive (RRV < 10), 36 isolates (73.47%) as low resistant (10 < RRV < 50), and 10 isolates (20.41%) as high resistant (RRV > 100). Isolate AHSA17 exhibited the highest EC_50_ value (266.33 µg/mL), with a corresponding resistance ratio value of 3012.77. A detailed breakdown of isolates from different geographic regions is presented in Table 1.

### 2.2. Point Mutations in Target Genes Conferring Boscalid Resistance

**Figure 2:**
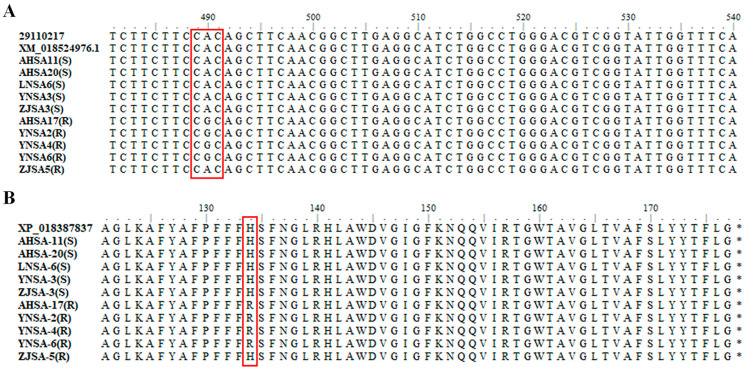
Sequence alignment of partial *sdhC* gene in five sensitive and five resistant isolates. (**A**) Sequences of nucleotides from loci 481 to loci 540 in *sdhC*. (**B**) Amino acid residue 121 to amino acid residue 178 in SDHC subunit. The codon and amino acid mutations are highlighted with red outlines. The “*” indicates the end of the amino acid sequence of SDHC.

The three genes (sdhB, sdhC and sdhD) encoding subunits of mitochondrial complex II were amplified and sequenced. Subsequent sequence alignment revealed no mutations in sdhB or sdhD. However, in resistant isolates AHSA17, YNSA2, YNSA4 and YNSA6, a DNA base transition from adenine to guanine at position 490 of the sdhC coding region was detected. This transition resulted in an amino acid substitution of histidine (CAC) to arginine (CGC) at amino acid position 134 (H134R). Conversely, no nucleotide alternations resulting in amino acid substitutions were identified in any of the three genes (sdhB, sdhC and sdhD) in the resistant isolate ZJSA5 (Figure 2). These findings suggest that the SDHC-H134R mutation is a primary determinant of boscalid resistance in Alternaria spp. isolated from strawberry.

### 2.3. Increased Mycelial Growth in Resistant Isolates

**Figure 3:**
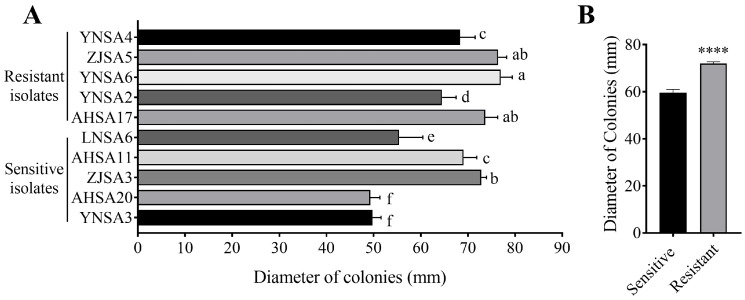
Mycelial growth. (**A**) Colony diameters of five sensitive and five resistant isolates after six-day culture on PDA plates. (**B**) Group comparison of mycelial growth for the sensitive and resistant populations (each for five isolates, *p* < 0.0001). Different lowercase letters above the columns indicate significant differences at *p* = 0.05 level. **** indicates significant difference at *p* = 0.0001 level.

Isolates exhibiting the highest five EC_50_ values and those with the lowest five EC_50_ values were selected for assessing mycelia growth on PDA without additional selection pressure. The results indicated that, with the exception of AHSA11 and ZJSA3 (Figure 3A), the five resistant isolates displayed faster mycelial growth rates compared to the sensitive isolates. Analysis of mean colony diameters revealed values of 59.28 ± 11.01 mm for the five sensitive isolates and 71.98 ± 5.41 mm for the five resistant isolates, demonstrating a significant increase in mycelial growth among resistant isolates (Figure 3B).

### 2.4. Conidia Production and Germination

**Figure 4:**
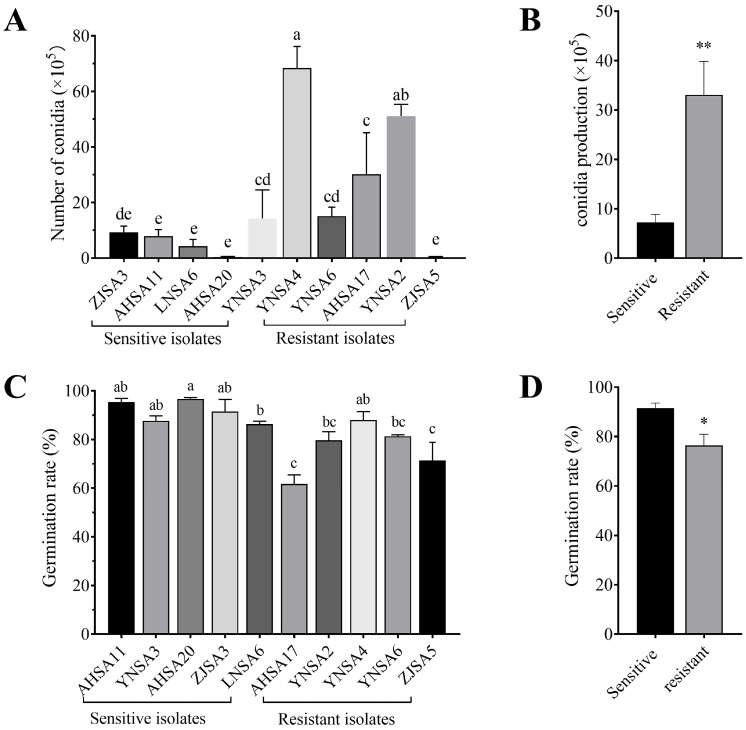
Conidia production and germination rate. (**A**) Number of conidia produced on PDA plates after 20-day culture at 25 °C. (**B**) Group comparison of conidia production for the sensitive and resistant populations (each for five isolates, *p* = 0.0055). (**C**) Conidial germination rates of five sensitive and five resistant isolates after 6 h incubation on WA medium. (**D**) Group comparison of conidial germination rates of the sensitive and resistant populations (each for five isolates, *p* = 0.0253). Different lowercase letters above the columns indicates significant difference at *p* = 0.05 level. * indicates significant difference at *p* = 0.05 level and ** indicates significant difference at *p* = 0.01 level.

Assessment of conidia production demonstrated that the five resistant isolates produced more conidia than the five sensitive isolates on PDA medium (Figure 4A). Group comparisons revealed that the resistant population exhibited an increased capacity for conidia production compared to the sensitive population (Figure 4B).

Conidial germination assays on WA medium showed that germination rates of the five resistant isolates were slightly lower than those of the five sensitive isolates (Figure 4C). Comparison of mean germination rates between the two groups revealed average mean germination rates of 91.50 ± 2.04% for the sensitive isolates and 76.40 ± 4.54% for the resistant isolates, indicating a significant decrease in conidial germination rate within the resistant population (Figure 4D).

### 2.5. Virulence

**Figure 5:**
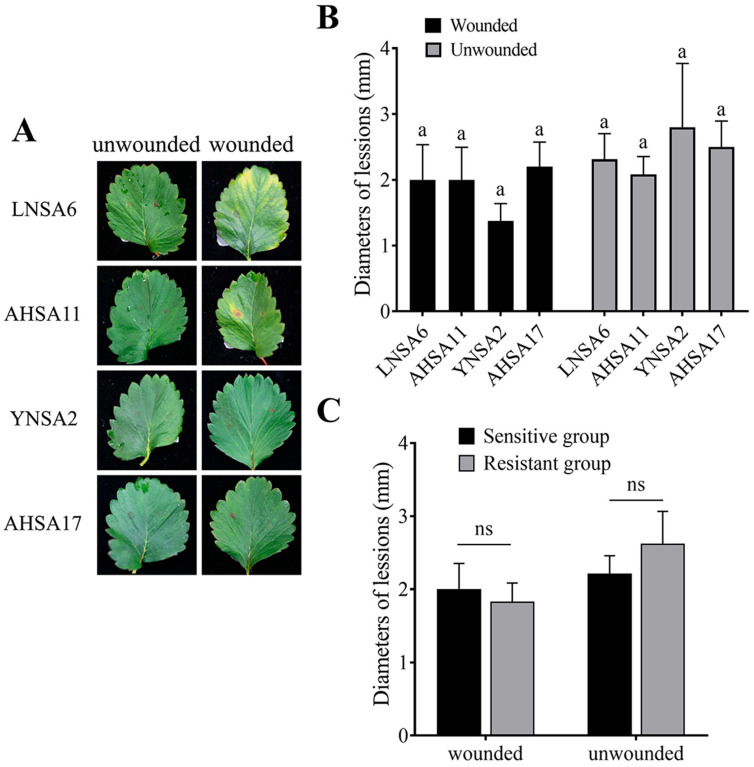
Virulence on strawberry leaves. (**A**) Lesions on strawberry leaves (sensitive isolates: LNSA6 and AHSA11, resistant isolates: YNSA2 and AHSA17). (**B**) Difference analysis of virulence by two-way ANOVA. Different lowercase letters above the columns indicate significant differences at *p* = 0.05 level. (**C**) Virulence analysis between resistant group and sensitive group. “ns” indicates no significant difference.

At 15 days post-inoculation, development of lesions on strawberry leaves was assessed. The results indicated that the average disease incidence was 90% on wounded leaves and 60% on unwounded leaves for the two resistant isolates (YNSA2 and AHSA17). In contrast, the average disease incidence was 85% on wounded leaves and 70% on unwounded leaves for the two sensitive isolates (LNSA6 and AHSA11). No significant difference in lesion diameter was observed between the sensitive and resistant isolates (Figure 5).

### 2.6. Responses to Stress Agents

**Figure 6:**
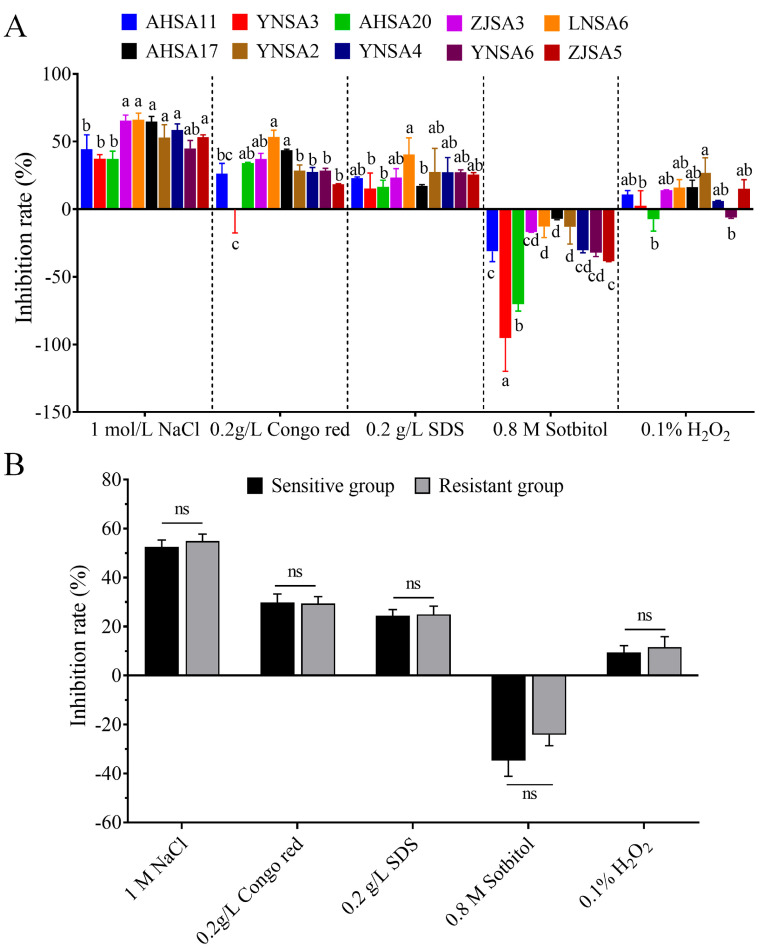
(**A**) Sensitivity to stress agents based on inhibition rate of mycelial growth on PDA medium supplemented with 1 M NaCl, 0.2 g/L Congo red, 0.2 g/L SDS, 0.8 M sorbitol or 0.1% H_2_O_2_, respectively. Different lowercase letters above the columns indicate significant differences at *p* = 0.05 level. (**B**) Analysis of difference between resistant group and sensitive group. “ns” indicates no significant difference.

The responses of five sensitive isolates and five resistant isolates to various stress agents were analyzed. No significant difference was detected in response to osmotic stress induced by 1 M NaCl between resistant and sensitive isolates. Regarding the response to carbohydrate stress, mycelial growth of all the isolates was stimulated by sorbitol, resulting in negative inhibition rates. Two sensitive isolates (YNSA3 and AHSA2) exhibited significant differences compared to the other isolates in their response to sorbitol. Furthermore, no significant differences were observed in the inhibition rate of Congo red and SDS, which disrupt cell wall and cell membrane integrity, respectively, between sensitive isolates and resistant isolates. Similarly, no significant difference was detected in the response to oxidative stress induced by H_2_O_2_ between sensitive and resistant isolates (Figure 6). The comparable ability of resistant and sensitive isolates to respond to diverse stress stimuli suggests that resistant isolates may exhibit similar survival rates compared to sensitive isolates in field conditions.

### 2.7. Cross-Resistance Between Boscalid and Other Fungicides

**Figure 7:**
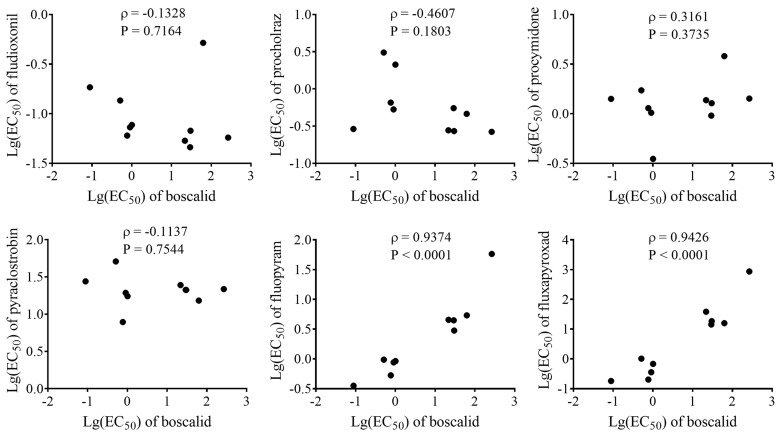
Cross-resistance between boscalid and six other fungicides (upper: left to right successively show the correlation of boscalid with fludioxonil, prochloraz and procymidone; lower: left to right successively show the correlation of boscalid with pyraclostrobin, fluopyram and fluxapyroxad, respectively). Positive cross-resistance between two different fungicides is indicated when Pearson’s correlation analysis showed ρ > 0.8 and *p* < 0.05.

Cross-resistance between boscalid and six other fungicides was investigated by assessing the correlation between the logarithms of EC_50_ value [lg(EC_50_)] for each fungicide pair. No cross-resistance was detected between boscalid (FRAC 7) and four fungicides with different modes of action: fludioxonil (FRAC 12), prochloraz (FRAC 3), procymidone (FRAC 2) and pyraclostrobin (FRAC 11). However, boscalid exhibited positive cross-resistance to fluopyram (FRAC 7), with a Pearson’s correlation coefficient (ρ) of 0.9374 (*p* < 0.0001). Similarly, positive cross-resistance was detected between boscalid and fluxapyroxad (FRAC 7) with a Pearson’s correlation coefficient (ρ) value of 0.9426 (*p* < 0.0001) (Figure 7).

### 2.8. SDHC-H134R Mutation Reduces the Binding Affinity of Boscalid to Mitochondrial Complex II

**Figure 8:**
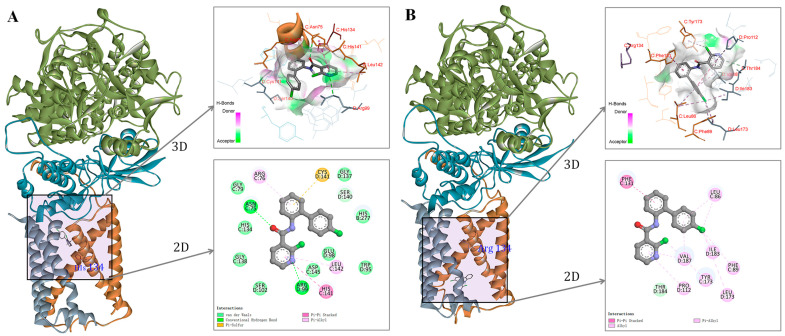
Molecular docking. (**A**) Docking result of boscalid to SDHC-134His model. (**B**) Docking result of boscalid to SDHC-134Arg model. The green, blue, gray and orange color indicate SDHA, SDHB, SDHC and SDHD subunits in the model respectively. The purple frame showed the region that enlarged to the 2D of 3D figures.

To examine the effect of SDHC-H134R mutation on the binding affinity of boscalid to mitochondrial complex II, CB-Dock analysis was performed to simulate the interaction of boscalid with the SDHC-134His or SDHC-134Arg models within the CB-Dock2 server (Figure 8). Docking results indicated that the Vina score for the boscalid-SDHC-134His complex was −8.6 kcal/mol, while the Vina score for the boscalid-SDHC-134Arg complex was −7.9 kcal/mol. Although boscalid did not directly interact with amino acid residue 134 of SDHC in either model, amino acid residue 134His of the SDHC subunit was located within the binding pocket in the SDHC-134His model, whereas amino acid residue 134Arg was not located within the binding pocket in the SDHC-134Arg model. Furthermore, hydrogen bonds were detected between boscalid with 75Asn of the SDHC subunit and 99Arg of the SDHD subunit in the SDHC-His134 model. In contrast, no hydrogen bonds were detected in the docking results for the SDHC-134Arg model. In summary, the molecular docking results suggest that the SDHC-H134R mutation reduced the binding affinity of boscalid to mitochondrial complex II in *Alternaria* spp.

## 3. Discussion

Fungal pathogens pose a significant threat to strawberry plants during growth and to fruit quality during postharvest transport and storage. Strawberry black spot, a disease caused by *Alternaria* spp. that affects both leaves and fruits, has been reported in several strawberry-growing regions in China and other countries. In this study, a collection of isolates obtained from symptomatic strawberry leaves were subjected to sensitivity analysis for boscalid, an SDHI fungicide that has been used for several years in China to manage strawberry diseases. The distribution frequency of EC_50_ values was investigated for these isolates, revealing the presence of resistant isolates. Furthermore, the survival fitness of resistant isolates was assessed and the underlying mechanisms of resistance were also analyzed.

Historically, strawberry disease management efforts have primarily focused on damage caused by *B. cinerea*, *F. oxysporum* and *Colletotrichum* species. However, strawberry black spot, caused by *Alternaria* spp., has been increasingly reported in some growing regions in recent years, potentially indicating a greater risk of *Alternaria* spp. causing damage to strawberry crops, particularly when susceptible cultivars are used. While limited research has specifically focused on this disease caused by *Alternaria* spp. in strawberry, numerous studies have assessed the sensitivity of *Alternaria* spp. to fungicides in other hosts. For example, mefentrifluconazole and pyraclostrobin have demonstrated excellent inhibition activity against *A. alternata* causing tomato early blight [17,26], and pyrimethanil, cyprodinil and fludioxonil also show good activity against *Alternaria* spp. causing potato early blight [27]. Furthermore, some SDHIs have exhibited good activity in inhibiting *Alternaria* spp. causing brown spot of citrus, black spot of pear and tobacco brown spot [18,28,29]. However, information about *Alternaria* spp. from strawberry is missing. In this study, we analyzed the sensitivity of 49 *Alternaria* spp. isolates collected from strawberry leaves to boscalid, revealing a wide range of EC_50_ values, from 0.0884 to 266.3289 µg/mL. Although boscalid is not registered to control strawberry black spot, it has been used for the control of strawberry diseases for several years and boscalid resistance of *B. cinerea* causing leaf and fruit rot has been reported in China [23,24]. The present study indicates that the extensive use of boscalid in strawberry production has also contributed to the development of boscalid resistance in *Alternaria* spp., which can cause incidental disease in strawberry (Figure 1). Despite the limited sample size, the proportion of resistant isolates reached 100% in Liaoning province (Table 1), highlighting the need for future research with larger sample sizes to monitor the sensitivity of *Alternaria* spp. from strawberry to boscalid in Liaoning. Furthermore, these findings emphasize the importance of considering the potential for resistance development in the casual agents of incidental diseases when implementing disease management strategies after prolonged use of a single fungicide. The detection of positive cross-resistance between boscalid and the SDHIs fluopyram and fluxapyroxad (Figure 7), which have also been registered to control strawberry disease in mixtures in China, suggests that SDHIs should be used judiciously in the management of strawberry diseases, particularly in single-product formulations. Furthermore, the absence of cross-resistance between boscalid and fludioxonil, prochloraz and pyraclostrobin suggests that fungicide mixtures containing these compounds may be a viable option for managing strawberry diseases. However, field trials are needed to confirm the efficacy of these combinations in controlling boscalid-resistant *Alternaria* populations. In regions with confirmed fungicide resistance, comprehensive surveys of existing spraying regimes are crucial before developing and implementing effective disease management strategies.

Resistance to SDHIs has been reported in *Alternaria* spp. from other hosts in the field. *A. alternata* causing black spot of pear has developed resistance to boscalid, associated with mutations SDHB-H277Y, SDHC-H134R and SDHD-D123E [30]. Mutations in SDHB, SDHC and SDHD subunits correlated with boscalid resistance have been identified in pathogens causing potato early blight in Europe and America; in addition, SDHC-H134R was the most prevalent mutation in *A. solani* and SDHB-H277Y was the predominant mutation in *A. alternata* [30,31]. In the present study, resistance to boscalid in *Alternaria* spp. from strawberry was found to be associated with the SDHC-H134R mutation, rather than other reported mutation types. Similarly, SDHC-H134R has been detected in *A. alternata* resistant to fluopyram and boscalid from pistachio [32,33], *A. solani* resistant to SDHIs from potato [34], and *A. alternata* resistant to boscalid from almond [35]. This shows the prevalence of SDHC-H134R mutations conferring SDHI resistance in *Alternaria* spp. Other mutations in *A. alternata* from pistachio, including B-H277R/L, B-P230A/R, B-N235D/T, C-S135R and D-H133R/P, have been linked to SDHI resistance [32,33]; however, these mutations were not detected in our assay. Furthermore, molecular docking analysis indicated that the SDHC-H134R mutation prevents amino acid residue 134 of SDHC from participating in the boscalid-binding pocket, thereby reducing the binding affinity of boscalid to mitochondrial complex II. The decreased affinity of boscalid to mitochondrial complex II further elucidates the resistance mechanism to boscalid in resistant isolates harboring the SDHC-H134R mutation. However, one resistant isolate, ZJSA5, did not exhibit any mutations in *sdhB*, *sdhC* and *sdhD* (Figure 2 and Figures S2–S4). To validate this finding, gDNA was re-extracted from ZJSA5 and fragments of *sdhB*, *sdhC* and *sdhD* were re-amplified and sequenced. Nonetheless, no mutations were detected. Similarly, three other resistant isolates (YNSA1, LNSA1 and AHSA22) did not show any mutations in *sdhB*, *sdhC* and *sdhD*, potentially indicating an alternative, yet unidentified, resistance mechanism. Therefore, further investigations should focus on these four isolates lacking mutations by re-sequencing their genomes or analyzing the expression of proteins involved in fungicide efflux.

Assessment of fitness is crucial for evaluating the survival capacity of resistant mutants. In our study, resistant isolates exhibited increased mycelial growth and higher conidia production compared to sensitive isolates (Figure 3B and Figure 4B). However, the conidial gemination rate was reduced in resistant isolates (Figure 4D). Additionally, no significant differences were observed in virulence and responses to stress agents between sensitive and resistant isolates (Figure 5 and Figure 6). Collectively, the decreased conidial germination rate in resistant isolates may be offset by increased conidia production and enhanced mycelial growth, suggesting that boscalid-resistant isolates of *Alternaria* spp. from strawberry exhibited minimal fitness costs. In parallel, *A. alternata* with the SDHC-H134R mutation causing black spot of pear, and *A. solani* resistant to SDHIs, have not shown evidence of significant fitness costs [30,34,36]. The absence of significant fitness costs may promote the proliferation of resistant isolates in field environments, particularly under the selection pressure imposed by a single fungicide. Therefore, implementing disease management strategies that combine fungicides with different modes of action is essential for disease control in the field. SDHI fungicides, especially those formulated as single-product treatments, should be used judiciously for the control of strawberry black spot in the future.

## 4. Materials and Methods

### 4.1. Isolates, Fungicides and Medium

A total of 49 *Alternaria* isolates were isolated from symptomatic strawberry leaves collected from greenhouses of Anhui, Liaoning, Yunnan and Zhejiang provinces during 2022–2023. Each isolate was purified by single conidia. All the isolates were determined as *Alternaria* spp. based on conidial morphology and stored at 4 °C in the laboratory. A stock solution of boscalid (10 mg/mL) was prepared by dissolving the fungicide in methanol. Potato dextrose agar (PDA) was used for general culture of isolates and for fungicide sensitivity testing with the exception of pyraclostrobin. Chemical-grade fludioxonil, prochloraz, procymidone, pyraclostrobin, fluxapyroxad and fluopyram were also dissolved in methanol to prepare 10 mg/mL stock solutions for use in cross-resistance tests. The purity and concentrations of all the fungicides are detailed in Table S1. AEA medium was used for cross-resistance assays involving pyraclostrobin. The formulations of PDA and AEA media have been described previously [37].

### 4.2. Sensitivity Assay for Boscalid

For preliminary testing, five randomly selected isolates (AHSA5, LNSA3, AHSA11, ZJSA1 and YNSA2) were used to determine boscalid sensitivity on PDA amended with serial concentrations of 0, 0.008, 0.04, 0.2, 1 and 5 µg/mL. Subsequently, all the 49 *Alternaria* spp. isolates were pre-incubated on PDA plates at 25 °C. After five days, mycelial plugs from colony margins were transferred to fresh PDA plates and cultured for an additional 5 days. Mycelial plugs of 5 mm diameter from colony margins were made and transferred onto the center of PDA plates amended with boscalid at the following concentrations: 0, 0.008, 0.04, 0.2, 1, 5 and 25 µg/mL. PDA plates were cultured at 25 °C in an incubator. After 6 days, colony diameters were measured in two approximately perpendicular directions and growth inhibition rates were calculated as described previously [38]. The effective concentrations inhibiting 50% of mycelial growth (EC_50_) were calculated using DPS 7.05. There are three replicates for each isolate at each boscalid concentration.

### 4.3. Amplification and Sequence Analysis of the Target Genes

Boscalid, a succinate dehydrogenase inhibitor (SDHI), targets succinate dehydrogenase (SDH) in mitochondrial complex II. The quinone binding site is composed of three subunits: iron–sulfur (SDHB), cytochrome bL (SDHC) and cytochrome bS (SDHD). Mutations in these three subunits have been consistently associated with SDHI resistance. To identify potential mutation sites in boscalid-resistant isolates, mycelia were harvested from five isolates exhibiting the lowest EC_50_ values and five isolates with the highest EC_50_ values. These isolates had been cultured on PDA plates for 5 days. Genomic DNA (gDNA) was then extracted from each isolate using the CTAB method. Nucleotide sequences of *sdhB* (gene id: 29110570), *sdhC* (gene id: 29110217) and *sdhD* (gene id: 29122160) were retrieved from the NCBI database and used as queries in a nucleotide BLAST search against the NCBI database. Highly similar sequences from *A. brassicae*, *Alternaria solani* and *A. alternata* were obtained and aligned using BioEdit. Based on the alignment results, three primer pairs (Table 2) were designed and synthesized by Beijing Tsingke Biotech Co. Ltd. (Beijing, China). These primers were used to amplify fragments of *sdhB*, *sdhC* and *sdhD*. PCR amplification was performed using Phanta Max Super-Fidelity DNA Polymerase (P505, Vazyme Biotechnology Co., Ltd., Nanjing, China), following the manufacture’s protocol. The resulting PCR products were sequenced by Tsingke Biotech Co., Ltd. (Beijing, China). Coding sequences were extracted from these assembled sequences based on the mRNA sequences of *sdhB* (XM_018524976.1), *sdhC* (XM_018524623.1) and *sdhD* (XM_018536566.1). These coding sequences were translated into amino acid sequences and aligned using the ClustalW multiple alignment method in BioEdit.

### 4.4. Biological Characterization of Sensitivity and Resistant Isolates

***Mycelial growth***: Five sensitive isolates exhibiting the lowest EC_50_ values (indicating sensitive isolates in this context) and five resistant isolates with the highest EC_50_ values (indicating resistant isolates in this context) were selected to analyze mycelial growth. All the isolates were pre-cultured on PDA plates for 5 days at 25 °C and then transformed onto fresh PDA plates for an additional 5 days of incubation. Mycelial plugs (5 mm in diameter) were taken from the margins of the colonies and transformed onto PDA plates. After 6 days of culture at 25 °C in the dark, colony diameter was measured in two approximately perpendicular directions and the mean colony diameter was calculated. Each isolate was tested with four replicates, and the experiment was performed twice.

***Sporulation and conidia germination***: Following the mycelial growth assessment, colonies were cultured continuously at 25 °C for an additional 14 days. Subsequently, 5 mL of sterile distilled deionized H_2_O (ddH_2_O) were added to each plate and mycelia were scraped from the colonies. The mycelial suspension was filtered through three layers of lens paper and centrifuged at 5000× *g* rpm for 5 min to pellet the conidia. After discarding the supernatant, the conidia were resuspended in 2 mL of ddH_2_O and the conidia concentration was determined using a hemocytometer. The total number of conidia produced per plate was then calculated.

After determining the number of conidia, the conidia suspension was adjusted to 1 × 10^5^ conidia per milliliter and 100 µL of the suspension was spread onto water agar (WA) medium. After 6 h of incubation at 25 °C, the germination rate of each isolate was determined by counting 100 conidia per plate using a microscope. Each isolate was tested with four replicates and the experiment was repeated twice.

***Virulence Assay***: Strawberry cultivar Jiandebailu was used in virulence assay. Conidia from two sensitive (AHSA11 and LNSA6) and resistant isolates (AHSA17 and YNSA2) were collected from 20-day-old cultures, as described previously. The conidia suspension was adjusted to 2 × 10^5^ conidia per milliliter. Twenty microliters of the conidial suspension were inoculated onto each inoculation site on detached strawberry leaves. The leaves were either wounded with pins or left unwounded prior to inoculation. Inoculated leaves were incubated at 25 °C with 85% relative humidity for 15 days, after which the lesions formed on the leaves were measured. Five leaves were inoculated for each isolate, with two inoculation sites per leaf. The experiments were independently performed twice.

### 4.5. Sensitivity to Stress Agents

Mycelial plugs from 5-day-old PDA cultures were taken from the margins of colonies and transferred onto PDA plates supplemented with 1 M NaCl, 0.2 g/L Congo red, 0.2 g/L SDS, 0.8 M sorbitol and 0.1% H_2_O_2_ to induce stress. After 6 days, colony diameters were measured in two approximately perpendicular directions. Inhibition rates were then calculated. Each treatment was performed with three replicates and the experiments were independently performed twice.

### 4.6. Cross-Resistance Analysis

Cross-resistance between boscalid and other fungicides was assessed using a mycelial growth inhibition assay. Five boscalid-sensitive isolates (YNSA3, AHSA20, ZJSA3, AHSA11 and LNSA6) and five boscalid-resistant isolates (YNSA4, ZJSA5, YNSA6, YNSA2 and AHSA17) were selected. The sensitivity of these isolates to fluxapyroxad, fluopyram, fludioxonil, prochloraz, procymidone and pyraclostrobin were determined. Fungicide concentrations are listed in Table S1. Three replicates were performed for each concentration of each fungicide.

### 4.7. Model Building and Molecular Docking

Amino acid sequences of SDHA (XP_018380410.1), SDHB (XP_018386725.1), SDHC (XP_018387837.1) and SDHD (XP_018380725.1) were retrieved from NCBI and submitted to SWISS-MODEL to construct protein models. The crystal structure of porcine heart mitochondrial complex II in complex with N-[(4-tert-butylphenyl)methyl]-2-(trifluoromethyl)benzamide (4YTP) was selected as a template for building the SDHC-134His model. This model exhibited 61.91% sequence identity to the template and a QMQE score of 0.71. An SDHC-134Arg model, incorporating a histidine-to-arginine substitution at position 134 of SDHC, was also generated using the same template, with 61.61% sequence identity and a QMQE score of 0.71. The three-dimensional structure of boscalid was obtained from the PubChem database (https://pubchem.ncbi.nlm.nih.gov/). Molecular docking of boscalid with the generated models was performed using AutoDock Vina within the CB-Dock2 server (https://cadd.labshare.cn/cb-dock2/php/index.php, accessed on 23 December 2024), focusing on a cavity encompassing residues of SDHC. The Vina score was utilized to evaluate the binding affinity between boscalid and each model [39]. Docking results were visualized using Discovery Studio 2019 Client.

### 4.8. Data Analysis

EC_50_ values were calculated using DPS software (version 7.05). For mycelial growth, conidia production and conidial germination assays, differences between mean values of resistant isolates and sensitive isolates were analyzed using a non-parametric *t*-test in GraphPad Prism 8.0. Virulence and inhibition rate of stress agents on mycelial growth were analyzed using two-way ANOVA in GraphPad Prism 8.0. Where multiple comparisons were performed, Tukey’s multiple comparison test was used. Pearson’s correlation coefficient (ρ) values were also calculated to assess the cross-resistance between two fungicides using GraphPad Prism 8.0.

## 5. Conclusions

This study suggested that resistance to boscalid has emerged in *Alternaria* spp. causing strawberry black spot in Chinese agricultural settings, and resistant isolates exhibited minimal fitness costs. The SDHC-H134R mutation confers resistance to boscalid, while an alternative, yet undeciphered, mechanism underlying boscalid resistance deserves further investigation. These findings provide valuable insights for developing effective strategies for managing strawberry black spot in the field.

## Figures and Tables

**Table 1:** Sensitivity to boscalid of isolates from different provinces.

Location	Number of Isolates	Range of EC_50_ (µg/mL)	EC_50_ (µg/mL)	Ratio of Resistant Isolates	Ratio of Highly Resistant Isolates
Anhui	29	0.5129–266.3289	11.9290 ± 9.1095	28/29	3/29
Liaoning	8	1.0068–13.4895	3.6321 ± 1.4727	8/8	1/8
Yunnan	6	0.0884–62.4968	25.0278 ± 8.5018	5/6	5/6
Zhejiang	6	0.7673–29.4114	6.5811 ± 4.5846	5/6	1/6

**Table 2:** Primers used in this study.

Primers	Sequence (5′-3′)	Purpose
B-SF	TGTCATAACCGAGGAAGC	Amplify a fragment of 1363 bp containing coding region of *sdhB*
B-SR	TAGATTGGCACAGGGAAC	
C-SF	CATAACGCCAGCAGACAA	Amplify a fragment of 1274 bp containing coding region of *sdhC*
C-SR	GATTCCCATAAACCACCC	
D-SF	GGGACGTCAGCAATCGACAT	Amplify a fragment of 937 bp containing coding region of *sdhD*
D-SR	CAGCAGGAAAGTGCTCCAAG	

## Data Availability

All additional datasets supporting the findings of this study are included within the article and Supplementary Materials.

## Supplementary Materials

The following supporting information can be downloaded at: https://www.mdpi.com/article/10.3390/plants14131941/s1, Figure S1: Sensitivity of *Alternaria* species to boscalid on PDA plates; Figure S2: Alignment of complete amino acid sequences in SDHB of *Alternaria* species, causing strawberry black spot; Figure S3: Alignment of complete amino acid sequences in SDHC of *Alternaria* species, causing strawberry black spot; Figure S4: Alignment of complete amino acid sequences in SDHD of *Alternaria* species, causing strawberry black spot; Table S1: Concentrations of fungicides used for sensitivity and cross-resistance tests.

## Abbreviations

The following abbreviations are used in this manuscript:

PDA	Potato dextrose agar
AEA	Acetic acid ester agar
SDHI	Succinate dehydrogenase inhibitor

## References

[1] Voća S., Šic Žlabur J., Dobričević N., Jakobek L., Šeruga M., Galić A., Pliestić S. (2014). Variation in the bioactive compound content at three ripening stages of strawberry fruit. Molecules.

[2] Sallato B.V., Torres R., Zoffoli J.P., Latorre B.A. (2007). Effect of boscalid on postharvest decay of strawberry caused by *Botrytis cinerea* and *Rhizopus stolonifera*. Span. J. Agric. Res..

[3] Lattanzio V., Di Venere D., Linsalata V., Lima G., Ippolito A., Salerno M. (1996). Antifungal activity of 2.5-dimethoxybenzoic acid on postharvest pathogens of strawberry fruits. Postharvest Biol. Technol..

[4] Henry P.M., Haugland M., Lopez L., Munji M., Watson D.C., Gordon T.R. (2020). The potential for *Fusarium oxysporum* f. sp. *fragariae*, cause of fusarium wilt of strawberry, to colonize organic matter in soil and persist through anaerobic soil disinfestation. Plant Pathol..

[5] Hu S.D., Zhang Y.T., Yu H., Zhou J.Y., Hu M.H., Liu A.C., Wu J.Y., Wang H.C., Zhang C.Q. (2022). *Colletotrichum* spp. diversity between leaf anthracnose and crown rot from the same strawberry plant. Front. Microbiol..

[6] Zhang Y.T., Yu H., Hu M.H., Wu J.Y., Zhang C.Q. (2022). Fungal pathogens associated with strawberry crown rot disease in China. J. Fungi.

[7] Wang Q.Z., Zhang S.K., Xu J.J., Ke X.Y., Peng C., Li Z., Chen B., Pan S., Gu T.T. (2022). Monitoring the infection of powdery mildew pathogen on strawberry leaves by ATR-IR technique. J. Pathol..

[8] Yuan S.Z., Wang B.G., Wang M., Sun M.M., Wang X.Q., Li X.F., Yang N., Xu X.D., Zheng S.F., Wang Q. (2024). Antifungal mechanism of protocatechuic acid methyl ester against *Botrytis cinerea* in postharvest strawberry fruit. Postharvest Biol. Technol..

[9] Fu Y., Zhang X.F., Liu S.J.H., Hu K.L., Wu X.H. (2020). Characterization of *Alternaria* species associated with black spot of strawberry in Beijing municipality of China. Can. J. Plant Pathol..

[10] Patil J.S., Suryawanshi N.S. (2014). Fruit rot of strawberry caused by *Alternaria alternata* control using homoeopathic medicines. Int. J. Pharm. Sci. Invent..

[11] Sun X.Z., Wang C.Y., Gao X., Wu X.H., Fu Y. (2023). Characterization of *Alternaria* species associated with black spot of strawberry in Dandong, China. Agronomy.

[12] Watanabe Y., Umekawa M. (1977). Black spot of strawberry caused by *Alternaria* spp.. Jpn. J. Phytopathol..

[13] Wada H., Cavanni P., Bugiani R., Kodama M., Otani H., Kohmoto K. (1996). Occurrence of the strawberry pathotypes of *Alternaria alternata* in Italy. Plant Dis..

[14] Cho J.T., Moon B.J. (1980). The occurrence of strawberry black leaf spot caused by *Alternaria alternata* (Fr.) Keissler in Korea. Korean J. Appl. Entomol..

[15] Mehmood N., Riaz A., Naz F., Hassan I., Jaabeen N., Anwaar S., Rosli H., Gleason M.L. (2018). First report of strawberry leaf spot caused by *Alternaria alternata* in Pakistan. Plant Dis..

[16] Bagherabadi S., Zafari D., Soleimani M.J. (2015). First report of leaf spot of strawberry caused by *Alternaria tenuissima* in Iran. J. Plant Pathol. Microbiol..

[17] Li G.X., Li X.H., Zeng Y., Liao S.L., Chen Y., Miao J.Q., Peng Q., Liu X.L. (2023). Three point mutations in AaCYP51 combined with induced overexpression of AaCYP51 conferred low-level resistance to mefentrifluconazole in *Alternaria alternata*. Pestic. Biochem. Phys..

[18] Liu Y.H., Dai D.J., Shen Y., Zhang C.Q. (2015). Detection of resistance of *Alternaria Kikuchiana* causing pear black spot to fungicides and baseline sensitivity of *A. kikuchiana* to boscalid. Chin. J. Pestic. Sci..

[19] Vegas B., Dewdney M.M. (2015). Sensitivity of *Alternaria alternata* from citrus to boscalid and polymorphism in iron-sulfur and in anchored membrane subunits of succinate dehydrogenase. Plant Dis..

[20] Sun J.Z., Ran W.Q., Feng L.C., Cui J., Fan W.Z., Pan Y.X., Zhao L.N. (2023). Pathogen identification of strawberry black spot and its susceptibility to fungicides. Agrochemicals.

[21] Al-Rahbi B.A.A., Al-Sadi A.M., Al-Mahmooli I.H., Al-Maawali S.S., Al-Mahruqi N.M.T., Celazhahan R. (2021). *Meyerozyma guilliermondii* SQUCC-33Y suppresses postharvest fruit rot of strawberry caused by *Alternaria alternata*. Australas. Plant Pathol..

[22] Demir S., Durak E.D., GÜneş H., Boyno G., Mulet J.M., Danesh Y.R., Procel R. (2023). Biological control of three fungal diseases in strawberry (*Fragaria* × *ananassa*) with arbuscular mycorrhizal fungi. Agronomy.

[23] Lin Z.S., Zhao S.F., Huang F.Y.H., Wu J.Y., Wang H.D., Zhang C.Q. (2018). Resistance to boscalid in *Botrytis cinerea* in Zhejiang Province. Chin. J. Pestic. Sci..

[24] Liu X., Wu Y., Cheng W., Zeng R., Xu H.L., Gao S.G., Dai F.M. (2018). Sensitivity and resistance mechanism to boscalid of *Botrytis cinerea* from strawberry in Shanghai. Chin. J. Pestic. Sci..

[25] Liu S.Y., Fan H.Y. (2024). The occurrence and mechanism of field resistance to boscalid and pyraclostrobin in *Stemphylium solani*, the causal agent of tomato gray leaf spot in China. Pestic. Biochem. Phys..

[26] Malandrakis A.A., Anastasios A., Apostolidou Z.A., Louka D., Markoglou A., Flouri F. (2018). Biological and molecular characterization of field isolates of *Alternaria alternata* with single or double resistance to respiratory complex II and III inhibitors. Eur. J. Plant Pathol..

[27] Budde-Rodriguez S., Pasche J.S., Mllik I., Gudmestad N.C. (2022). Sensitivity of *Alternaria* spp. from potato to pyrimethanil, cyprodinil and fludioxonil. Crop Prot..

[28] He M.X., Fu Y.S., Ruan R.X., Li H.Y. (2016). Sensitivity assay of *Alternaria alternata* from citrus in China to four new fungicides. J. Zhejiang Univ. (Agric. Life Sci.).

[29] Lei F.B., Wang H.C., Dai Y.F., Zhang C.Q. (2021). Sensitivity to boscalid of *Alternaria alternata* causing tobacco brown spot in Guizhou province. Chin. J. Pestic. Sci..

[30] Landschoot S., Carrette J., Vandecasteele M., De Baets B., Hofte M., Audenaert K., Haesaert G. (2017). Boscalid-resistance in *Alternaria alternata* and *Alternaria solani* populations: An emerging problem in Europe. Crop Prot..

[31] Miles T.D., Fairchild K.L., Merlington A., Kirk W.W., Rosenzweig N., Wharton P.S. (2013). First Report of boscalid and penthiopyrad-resistant isolates of *Alternaria solani* causing early blight of potato in Michigan. Plant Dis..

[32] Avenot H.F., Luna M., Michailides T.J. (2019). Phenotypic and molecular characterization of resistance to the SDHI fungicide fluopyram in populations of *Alternaria alternata* from pistachio orchards in California. Crop Prot..

[33] Avenot H.F., Michailides T.J. (2020). Occurrence and extent of boscalid resistance in populations of *Alternaria alternata* from California pistachio orchards. Plant Dis..

[34] Bauske M.J., Mallik I., Yellareddygari S.K.R., Gudmestad N.C. (2018). Spatial and temporal distribution of mutations conferring QoI and SDHI resistance in *Alternaria solani* across the United States. Plant Dis..

[35] Förster H., Luo Y., Hou L.L., Adaskaveg J.E. (2022). Mutations in *Sdh* gene subunits confer different cross-resistance patterns to SDHI fungicides in *Alternaria alternata* causing *Alternaria* leaf spot of almond in California. Plant Dis..

[36] Fan Z., Yang J.H., Fan F., Luo C.X., Schanabel G. (2015). Fitness and competitive ability of *Alternaria alternata* field isolates with resistance to SDHI, QoI and MBC fungicides. Plant Dis..

[37] Li T., Xu J.K., Gao H., Cao Z.G., Wang J.X., Cai Q.Y., Duan Y.B., Zhou M.G. (2022). The G143A/S substitution of mitochondrially encoded cytochrome b (Cytb) in *Magnaporthe oryzae* confers resistance to quinone outside inhibitors. Pest. Manag. Sci..

[38] Li T., Li N., Lei Z.Y., Zhang C.Q. (2023). Sensitivity and resistance risk of *Botryosphaeria dothidea* causing Chinese hickory trunk canker to fludioxonil. Pest. Biochem. Phys..

[39] Liu Y., Yang X.C., Gan J.H., Chen S., Xiao Z.X., Cao Y. (2022). CB-Dock2: Improved protein-ligand blind docking by integrating cavity detection, docking and homologous template fitting. Nucleic Acid. Res..

