# Supplementary material for: Resistance of Alternaria spp. Causing Strawberry Black Spot to Boscalid in China
Source: Plants (Basel). 2025 Jun 24;14(13):1941. doi: 10.3390/plants14131941 (PMC12251906; doi:10.3390/plants14131941)
Supplement: Supplementary file 1 [file plants-14-01941-s001.zip › plants-3669434-supplementary.pdf]

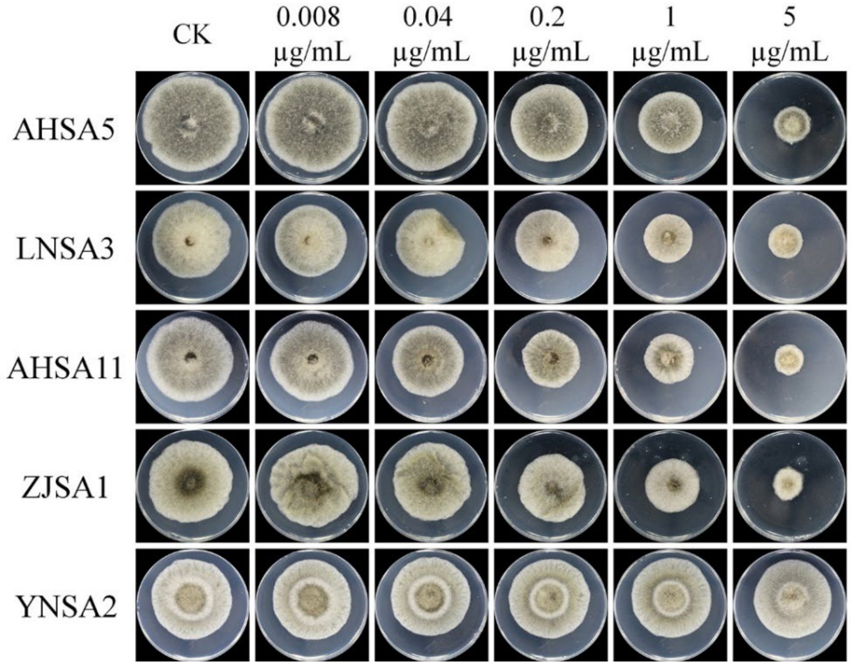

Figure S1 Sensitivity of *Alternaria* species to boscalid on PDA plates

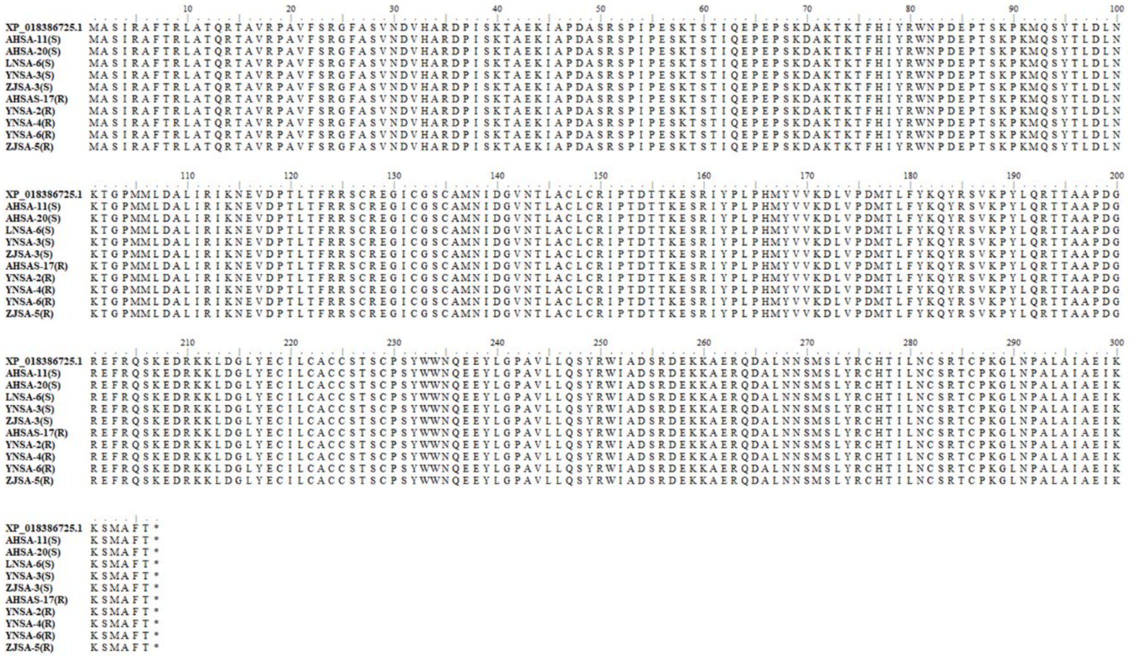

Figure S2 Alignment of complete amino acid sequences in SDHB of *Alternaria* species, causing strawberry black spot

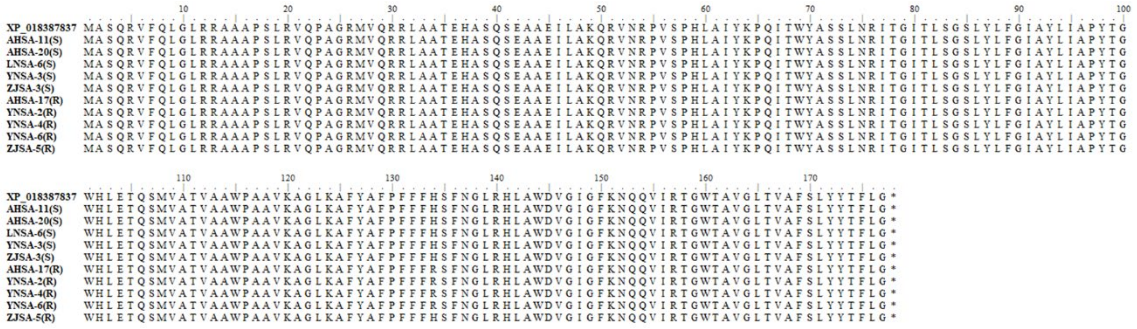

Figure S3 Alignment of complete amino acid sequences in SDHC of *Alternaria* species, causing strawberry black spot

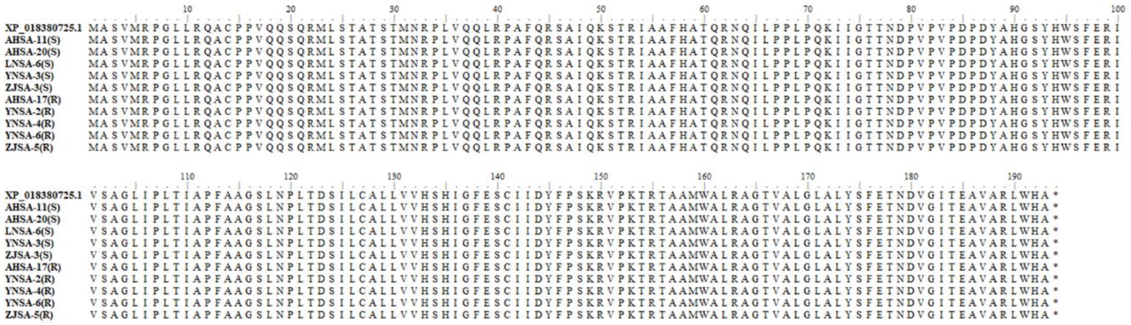

Figure S4 Alignment of complete amino acid sequences in SDHD of *Alternaria* species, causing strawberry black spot

Table S1 Concentrations of fungicides used for sensitivity and cross-resistance test

Fungicide	Purify of active ingredient	Manufacturers	Concentration (μg/mL)
Boscalid	97%	Jiangxi Zhengbang Crop Protection Co., Ltd	0, 0.008, 0.04, 0.2, 1, 5, 25
Fludioxolil	95%	Jiangxi Zhengbang Crop Protection Co., Ltd	0, 0.05, 0.1, 0.2, 0.4, 0.8
prochloraz	97%	Zhejiang Tianfeng Bioscience Co. Ltd	0, 0.00625, 0.025, 0.1, 0.4, 1.6
procymidone	95%	Zhejiang Tianfeng Bioscience Co. Ltd	0, 0.25, 0.5, 1, 2, 4, 8
pyraclostrobin	98.01%	Zhejiang Xinnong Chemical Co., Ltd	0.08, 0.4, 2, 10, 50
fluxapyroxad	99.6%	BASF (China) Co., Ltd	0, 0.032, 0.16, 0.8, 4, 20
fluopyram	96.4%	BASF (China) Co., Ltd	0, 0.032, 0.16, 0.8, 4, 20
